# Revising the reproductive story: psychosocial and reproductive impacts 12 months after reproductive genetic carrier screening

**DOI:** 10.1038/s41431-025-01903-z

**Published:** 2025-07-09

**Authors:** Erin Tutty, Belinda J. McClaren, Sharon Lewis, Kristine Barlow-Stewart, Tiffany Boughtwood, Jade Caruana, Jane L. Halliday, Edwin P. Kirk, Nigel G. Laing, John Massie, Martin B. Delatycki, Alison D. Archibald

**Affiliations:** 1 Department of Paediatrics, The University of Melbourne, Parkville, VIC Australia; 2 Genomics in Society, Murdoch Children’s Research Institute, Parkville, VIC Australia; 3 Reproductive Epidemiology, Murdoch Children’s Research Institute, Parkville, VIC Australia; 4 Faculty of Medicine and Health, Northern Clinical School, University of Sydney, St Leonards, NSW Australia; 5 Faculty of Medicine and Health, University of New South Wales, Sydney, NSW Australia; 6 Australian Genomics, Parkville, VIC Australia; 7 Genomics Health Alliances, Murdoch Children’s Research Institute, Parkville, VIC Australia; 8 Translational Genomics, Murdoch Children’s Research Institute, Parkville, VIC Australia; 9 Centre for Clinical Genetics, Sydney Children’s Hospital, Randwick, NSW Australia; 10 NSW Health Pathology Randwick Genomics Laboratory, Randwick, NSW Australia; 11 School of Clinical Medicine, University of New South Wales, Randwick, NSW Australia; 12 Centre for Medical Research, University of Western Australia, Nedlands, WA Australia; 13 Harry Perkins Institute of Medical Research, Nedlands, WA Australia; 14 Respiratory, Murdoch Children’s Research Institute, Parkville, VIC Australia; 15 Department of Respiratory Medicine, The Royal Children’s Hospital, Parkville, VIC Australia; 16 Children’s Bioethics Centre, The Royal Children’s Hospital, Parkville, VIC Australia; 17 Victorian Clinical Genetics Service, Parkville, VIC Australia; 18 Bruce Lefroy Centre, Murdoch Children’s Research Institute, Parkville, VIC Australia

**Keywords:** Social sciences, Genetics

## Abstract

The responsible implementation of reproductive genetic carrier screening (RGCS) involves understanding the long-term psychosocial and reproductive impacts of results. This mixed-methods study examined these impacts within ‘Mackenzie’s Mission’, an Australia-wide study that offered couple-based RGCS for >1280 genes to 10,000 reproductive couples. Data from participant surveys completed at enrolment and 12 months post-result were analysed. Participants with an increased chance result were interviewed. Reflexive thematic analysis, guided by Interpretive Description was used. 4948 participants (27% response) completed the 12 month post-result survey. Most had minimal decision regret (median ≤5, 0 = no regret, 100 = high regret) and high reproductive confidence. Participants found to have an increased chance result had elevated anxiety (*n* = 116, median = 39 out of 80, clinically meaningful is ≥40). Interviewees (*N* = 19, from 16 couples) felt their increased chance result *“change[d] everything”* about their reproductive plans. Although revising their reproductive plan was an emotionally complex *“journey”*, participants were *“grateful”* for the information. The concept of the ‘Reproductive Story’, was used to interpret the results. A reproductive story refers to a person’s expected narrative about parenthood that, if altered, can cause psychosocial distress. Receiving an increased chance result disrupts the reproductive story. By 12 months post-result, most people with an increased chance result felt empowered to revise their reproductive story, but anxiety was elevated. Findings suggest a need for longitudinal models of post-RGCS psychosocial support.

## Introduction

Reproductive genetic carrier screening (RGCS) provides people with information about their chance of having children with autosomal recessive or X-linked genetic conditions. Approximately 2% of reproductive couples (i.e. the two genetic parents, of opposite chromosomal sex, to a current or planned pregnancy) will have an increased chance of having children with a serious inherited condition, resulting in a 25% chance of the genetic condition in any pregnancy conceived (referred to hereafter as ‘increased chance result’) [1]. Many professional organisations now recommend that RGCS be offered to all people pre-conception or in early pregnancy, regardless of family history [2–5]. Two important aspects of population-wide implementation of RGCS are supporting reproductive autonomy and supporting people who receive increased chance results [6]. Thus, it is critical to understand the psychosocial and reproductive impacts of RGCS.

Most people expect to receive a low chance RGCS result, with results typically associated with peace-of-mind and increased reproductive confidence [7, 8]. In contrast, people are often under-prepared to receive an increased chance result. Common reactions to result disclosure include shock, anxiety, and fear [8–11]. The subsequent reproductive decision-making process is also known to be complex and psychosocially impactful [12, 13].

Couples with an increased chance result have access to several reproductive options, although options vary based on geographical location [14]. Nevertheless, for those with access to all available options, most still describe choosing between in-vitro fertilisation (IVF) with pre-implantation genetic testing for the monogenic condition (PGT-M) and prenatal diagnosis (PND) with the view to end an affected pregnancy [12, 13, 15–17]. Across three studies from Australia and the United States, more than 60% of people chose to use one of these reproductive interventions to avoid the genetic condition in their children [15–17]. Research has typically captured reproductive choices, or intended choices, for people’s first pregnancy at or soon after RGCS. Little research has explored how increased chance results impact reproductive plans throughout the reproductive stage-of-life.

There is some evidence to indicate that with time, people report feeling empowered by the information [8, 10, 18, 19]. However, heterogeneity in data collection methods, varied lengths of follow-up, and small sample sizes make it difficult to adequately assess the long-term psychosocial and reproductive impacts of RGCS [20].

### Study aim

The aim of this study was to explore experiences of RGCS within the first 12 months post-result, including:

1. Psychosocial outcomes before and 12 months after receiving RGCS results.
2. How an increased chance result impacts reproductive plans.

## Methods

### Study setting

This study was part of The Australian Reproductive Genetic Carrier Screening Project (‘Mackenzie’s Mission’), which offered RGCS free-of-charge, before or in early pregnancy, for 1281 autosomal recessive and X-linked genes associated with ~750 serious childhood-onset conditions [1, 21]. Both reproductive partners were screened together and received a combined result [1]. The socio-demographics of the cohort generally reflected that of Australians of reproductive age [1]. Of 9107 reproductive couples screened, almost 2% (*n* = 175) of couples were newly identified through participation in Mackenzie's Mission to have an increased chance of having children with a condition screened (‘new’ increased chance, including four who already knew they had an increased chance for a different condition. See Kirk et al. [1] for a list of conditions.). A further 176 couples knew, prior to Mackenzie’s Mission, that they had an increased chance for one of the conditions screened and received a low chance for all other conditions (‘known’ increased chance) [1]. New increased chance results were disclosed by a study genetic counsellor and offered genetic counselling to discuss their reproductive options. In Australia, prenatal diagnosis and termination of pregnancy are available through the public health system. IVF with PGT-M is primarily available on a user-pay basis; to assess uptake in the absence of cost, couples screened pre-conception were offered one funded cycle of IVF with PGT-M [21]. Research was conducted alongside the RGCS program; this study draws on data collected at enrolment and 12 months post-result [21].

### Study design

This study used an explanatory sequential mixed-methods approach, enabling qualitative data to explain, and provide richness to, a quantitative dataset [22]. The qualitative enquiry was guided by Interpretive Description, a flexible methodological framework that draws upon the constructivist paradigm commonly used in qualitative research to develop findings applicable to clinical practice [23].

### Data collection

#### Survey

Demographics and baseline psychosocial data were collected from all participants at enrolment. The 12 month post-result survey was optional. State anxiety was measured at both timepoints using the short form of the State Trait Anxiety Inventory (STAI-6) [24]. Scores range from 20 to 80 with scores ≥40 considered clinically meaningful [25]. The 12 month post-result survey also included the Decision Regret Scale (DRS, scores ranging from 0 = no regret to 100 = high regret [26]) and purpose-designed questions to capture reproductive confidence and reproductive choices.

#### Interviews

Participants with a new increased chance result were offered an interview, regardless of 12 month post-result survey completion. Participants were purposively sampled [27] to ensure the experiences of a variety of reproductive choices were captured. Reproductive intentions and outcomes data, which couples had already provided to Mackenzie’s Mission [1], were used to aid sampling. At the time of sampling, couples were either undecided or had chosen one of the following pathways: IVF with PGT-M, PND with the view to end an affected pregnancy, using their increased chance result to plan/prepare to potentially have a child with the genetic condition or not having (more) children [1].

Both members of a couple were invited to take part and if both accepted, separate interviews were organised. This approach allowed for in-depth engagement with each participant, and they could discuss potentially sensitive or emotionally complex topics, such as relationship dynamics, without the influence of the other partner. Semi-structured interviews were conducted over telephone and audio-recorded, with participants providing verbal consent to participate. Audio-recordings were transcribed verbatim and de-identified. Participants were assigned pseudonyms; if both partners participated, pseudonyms beginning with the same letter were selected.

### Data analysis

#### Survey

Demographic data were described using frequencies. To assess possible response bias at 12 months post-result, the demographics of responders and non-responders were examined using X^2^ tests. STAI, DRS and reproductive confidence scores were summarised per-individual, then grouped by the RGCS result received. Reproductive choices and psychosocial impacts for participants with a new increased chance result were summarised based on their reproductive status (e.g., currently pregnant).

#### Interviews

Interviews were analysed using reflexive thematic analysis, a form of qualitative analysis that involves distilling data into shared patterns of meaning [28]. Analysis began with data familiarisation followed by inductive coding. If both members of a couple participated, transcripts were compared to examine participants’ unique experience of the same reproductive journey, with similarities and differences noted. As analysis progressed, the study team met to discuss codes, and the development and refinement of themes. This iterative, team-based approach to reflexive thematic analysis meant findings were developed from multiple viewpoints, increasing interpretive rigour.

## Results

### Survey

**Fig. 1:**
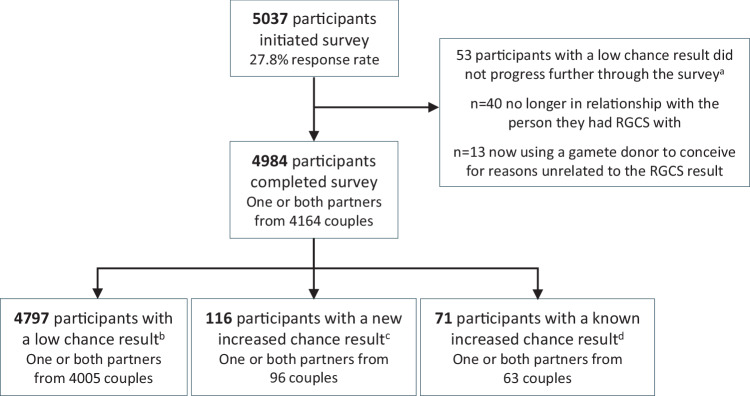
Survey participation. RGCS = reproductive genetic carrier screening | ‘New’ increased chance = People newly identified through Mackenzie’s Mission as having an increased chance of having children with a condition screened | ‘Known’ increased chance = people who already knew they had an increased chance of children with an inherited condition prior to taking part in Mackenzie’s Mission and received a low chance for all other conditions screened in the study. ^a^Participants were not asked any further survey questions as the combined low chance result was no longer relevant for reproductive planning ^b^For low chance results, 27.4% individual response rate with 45.7% of couples represented. ^c^For new increased chance results, 33.1% individual response rate with 54.9% of couples represented ^c^ For known increased chance results, 20.3% individual response rate with 35.8% of couples represented.

Responses to the 12 month post-result survey from 4984 participants, including 116 with a new increased chance result, were analysed (see Fig. 1). Characteristics are described in Table 1. Of note, 71% of participants were female. Further, at enrolment in MM, 83% of couples were not pregnant and 60% of participants were planning a pregnancy in the next year.

**Table 1:** Survey participant characteristics as reported at study enrolment^a^.

Couple characteristics^b^	*N* (%)
Socioeconomic status ranking of area lived in^c^	
Quintile 1 (most disadvantaged areas)	251 (6.0)
Quintile 2	552 (13.3)
Quintile 3	779 (18.7)
Quintile 4	991 (23.8)
Quintile 5 (most advantaged areas)	1591 (38.2)
Language spoken at home	
English only	3696 (88.8)
English and other language(s)	349 (8.4)
Language(s) other than English	119 (2.9)
Relevant family history of a genetic condition^d^	399 (9.6)
Pregnant at enrolment in Mackenzie’s Mission	691 (16.5)
Number of children	
Zero	3122 (75)
One	783 (18.8)
Two or more	259 (6.2)
Has a child a with medical condition/disability	234 (5.6)
Reproductive history	
Experienced a miscarriage	832 (20.0)
Experienced a stillbirth	123 (3.0)
Experienced a termination of pregnancy	512 (12.3)
Individual characteristics	*N* (%)
Gender	
Female	3562 (71.5)
Male	1419 (28.5)
Prefer not to say	3 (0.1)
Born in Australia	3839 (77.0)
Affinity with a religion	
Yes	1964 (39.4)
No	2970 (59.6)
Prefer not to say	50 (1.0)
Influence of religion on life decisions	
Not at all	3498 (70.2)
Very little	762 (15.3)
Moderately	500 (10.0)
Very much	158 (3.2)
Completely	66 (1.3)
Highest level of education attained	
Bachelor degree or above	3958 (79.4)
Advanced diploma or diploma	123 (2.4)
Certificate	597 (11.9)
High school or below	306 (6.1)
Intention to conceive in the future	
In the next year	2515 (60.3)
In more than one year	1174 (28.1)
Unsure	367 (8.8)

^a^To assess possible response bias, characteristics of responders and non-responders were compared using X^2^ tests (see Supplementary Table 1).

^b^If both members of a couple completed the survey, responses from the female partner were reported.

^c^As per the Index of Relative Socioeconomic Advantage and Disadvantage.

^d^The couple has a family history of a genetic condition screened in Mackenzie’s Mission.

Psychosocial outcomes are described in Table 2. Median state anxiety was at, or close to, clinically meaningful levels for participants with an increased chance (both new and known) at 12 months post-result. For participants with a new increased chance, the highest levels of anxiety were seen amongst those who were currently trying to become pregnant, were planning a future pregnancy and/or had experienced a pregnancy loss in the last 12 months (see Supplementary Table 2 for reproductive choices made by couples with a new increased chance result). Median decision regret was zero for participants with a low chance result and a known increased chance result (IQR = 0–10) and five (IQR = 0–15) for participants with a new increased chance result. Reproductive confidence was highest for those with a low chance result, although 41% of participants with a new increased chance result also felt the result provided “a lot of confidence” when planning pregnancies.

**Table 2:** Anxiety, decision regret and reproductive confidence at 12 months post-result.

	Result^a^		
	Low	New increased chance	Known increased chance
*N*	4797	116	71
State anxiety^b^ at enrolment (median, IQR)	30 (23.3–36.7)	30 (23.3–40.0)	33.3 (26.7–43.3)
State anxiety at 12 months post-result (median, IQR)	26.6 (20–36.7)	38.3 (26.7–50.0)	40 (33.3–50.0)
Change in state anxiety (mean, 95% CI)	−2.2 (−2.5–−1.8)	6.8 (4.2–9.5)	5.0 (1.6–8.4)
State anxiety ≥40 at enrolment (*N*, %)	1139 (23.7)	36 (31)	27 (38)
State anxiety ≥40 at 12 months post-result (*N*, %)	1042 (21.3)	58 (50.0)	42 (59.2)
Decision regret^c^ (median, IQR)	0 (0–10)	5 (0–15)	0 (0–10)
Reproductive confidence (*N*, %)			
No confidence	16 (0.3)	1 (0.9)	4 (5.6)
Not much confidence	47 (1.00)	8 (6.9)	9 (12.7)
Some confidence	1441 (30.0)	59 (50.9)	21 (29.6)
A lot of confidence	3293 (68.6)	48 (41.4)	37 (52.1)

*IQR* interquartile range.

^a^‘New’ increased chance refers to people newly identified through Mackenzie’s Mission as having an increased chance of having children with a condition screened. ‘Known’ increased chance refers to people who already knew they had an increased chance of children with one of conditions screened prior to taking part in Mackenzie’s Mission and received a low chance for all other conditions screened in the study.

^b^Anxiety measured by the 6-item State Trait Anxiety Inventory (score range 20-80, higher scores indicate higher anxiety) [29]. Scores ≥40 indicate clinically meaningful anxiety [30].

^c^Decision regret measured by the Decision Regret Scale (scores range from 0-100, higher scores indicate higher anxiety) [31].

### Interviews

Nineteen participants with a new increased chance result (12 females and 7 males), including both partners from three couples, took part. The reproductive histories and reproductive choices of the 16 couples are presented in Table 3. Four themes were developed, describing the evolving psychosocial impacts of an increased chance result experienced within the first 12 months post-result. Illustrative quotes are provided in-text and in Table 4.

**Table 3:** Reproductive histories and reproductive choices of interviewees (*N* = 16).

	*N*
Number of children at enrolment	
None	13
One	3^a^
Reproductive history at enrolment	
Miscarriage	3
Termination of pregnancy	3
Reported difficulties conceiving at enrolment	
No	4
Yes - Haven’t used fertility treatment	2
Yes - Have used fertility treatment	2
Haven’t tried to conceive	8
Reproductive status at 12 months post-result	
No pregnancies during the study period	7
Pregnant at result disclosure	3
Became pregnant after result disclosure	6
Intended reproductive choice for couples who have not had a pregnancy during the study^b^	
PND^c^	1
IVF with PGT-M	5
Undecided	1
Reproductive choice for a couple’s first pregnancy during the study^b^	
PND^c^	6^d^
Testing after birth	1
IVF with PGT-M	2
Intended future reproductive choice for couples who had a pregnancy during the study	
IVF with PGT-M	1
Not having more children	2
Undecided	6

*PND* prenatal diagnosis, *IVF with PGT-M* in vitro fertilisation with pre-implantation genetic testing for the monogenic condition

^a^Two children born to couples prior to enrolment in the study underwent genetic testing after their parents received their increased chance result and were found not to have the condition. A third child did not undergo genetic testing as, based on the phenotype of the condition, it would have already been apparent if they were affected.

^b^Interviewees were sampled based on the reproductive intentions and outcomes data already provided to the Mackenzie’s Mission study. Whilst Mackenzie’s Mission participants had access to all available reproductive options, none had chosen to use donor gametes or foster/adopt a child.

^c^PND is used to refer to people who became pregnant spontaneously or with IVF without the use of PGT-M, and had diagnostic testing with the view to end an affected pregnancy.

^d^3/6 couples who had PND were pregnant at result disclosure. All six pregnancies were not affected by the genetic condition.

**Table 4:** Illustrative quotes from interview participants who received an increased chance result.

#	Quote
1	*“…it* [RGCS] *was just more of a check box…* [a] *double check that this is all good. So, I wasn’t too stressed about it, getting the results, because I just didn’t expect anything.”* – Naomi, pregnant post-result, had PND and pregnancy was unaffected
2	*“Actually, waiting for them* [the results], [there] *wasn’t that much anxiety because I was expecting them to come back as normal. It was…when the results did come back and they weren’t, and we were already pregnant, that I was a bit upset.”* – Camille, pregnant at result disclosure, had PND and pregnancy was unaffected
3	*“I do think we spoke about it once or twice, but in our mind…it just made sense* [to have PND]…*We’d be better to find out and make an informed decision* [about whether to continue the pregnancy]…” – Vera, pregnant at result disclosure, had PND and pregnancy was unaffected
4	*“…we spoke to some close friends…our parents as well* [about IVF with PGT-M]. *Not that we would have changed our mind if they had not been* [supportive]*…we just wanted to know what people’s ideas and perceptions around it were so that we could actually make our final decision.”* – Carlos, conceived post-result via IVF with PGT-M
5	*“I felt it* [having a child with the genetic condition] *was something that would be quite manageable for us, so it didn’t impact our decision to continue trying to get pregnant…”* – Leah, pregnant at the interview and will have testing for the condition immediately after birth
6	*“I think it’s also being prepared for the trauma of both options, because we might also then go through IVF and not get enough embryos…but there’s also trauma going through naturally.” –* Tilly, increased chance result, currently deciding on a reproductive option
7	*“…it was really hard…the whole first trimester where it was like ‘maybe we’re pregnant, maybe we’re not’. Not that we weren’t pregnant, we were, but it was ‘maybe it will end up as a full pregnancy or maybe it won’t’.”* – Naomi, became pregnant post-result, had PND and pregnancy was unaffected
8	*“…it* [the RGCS result] *was on my mind a lot after that* [PND result showing that the pregnancy was unaffected]…*probably the anxiety of the initial three months and then…there’s always that thought that maybe it’s* [the diagnostic result] *wrong…”* – Gabby, became pregnant post-result, had PND and pregnancy was unaffected
9	*“I* [saw] *other ladies, they’re very happy they’re pregnant and me, I was happy I was pregnant but at the same time…I was worried.”* – Samira, pregnant post-result, organised testing after birth and baby was unaffected
10	*“I just felt a little bit guilty because I was the one doing virtually nothing. In terms of all the injections and all the hormonal disruptions and having to go in for the ultrasounds…it was more about just supporting my wife.”* – Rory, conceived post-result via IVF with PGT-M
11	*“*[I will be] *quite excited if it* [IVF] *works, but…it’s almost a coin toss as to whether it works or not, but I think we just have to see how it goes…”-* Felix, planning to conceive via IVF with PGT-M
12	*“…the joke that people still keep saying, ‘Oh, you guys might have a whoopsie’* [unexpected pregnancy]*…that triggers you and your body. A fight or flight response comes back to that initial phone call* [from the genetic counselor]. [People] *don’t realise that you should never comment on that sort of thing… That’s a big “Oh yeah, no, no, no, we can’t have a whoopsie.” We cannot because it means us either getting an abortion or going through to 20 weeks to go through all of this again.”* – Vera, pregnant at result disclosure, had PND and the pregnancy was unaffected
13	*“Probably* [thinking about pregnancy] *a bit earlier than we would have…our baby’s only 10 months old and we’re not ready right this minute…but because it seems like there’s a little bit of a wait with some of the testing and things that we need to do beforehand* [for IVF with PGT-M]…” – Mara, pregnant post-result, had PND and pregnancy was unaffected

*RGCS* reproductive genetic carrier screening, *PND* prenatal diagnosis, *IVF with PGT-M* in vitro fertilisation with pre-implantation genetic testing for the monogenic condition.

***Theme 1 –*** **Receiving an increased chance result alters the expected reproductive path**

Most interviewees expected to receive a low chance result. Most who were offered RGCS pre-conception were hoping to achieve peace of mind before becoming pregnant (Table 4, quote 1). Other participants considered receiving an increased chance result to be unlikely and were unconcerned about trying to conceive whilst waiting for results (quote 2). As the increased chance result was unexpected, result disclosure appeared to have a profound emotional effect. Twelve months on, recollections remained vivid and descriptive.

*“It was very difficult to process…it certainly wasn’t far off…hearing of the death of a relative. It was that sort of level of emotion.”* – Adrian, increased chance, planning to conceive via IVF with PGT-M

Most participants described feeling that an increased chance result had profoundly altered their reproductive story. As Felicity stated, *“I just remember thinking… this is going to change everything.”* Participant narratives also indicated a sense of disempowerment in their reproductive futures and for some, the result challenged core beliefs about their lives.

*“…We just thought ‘oh, everything will be fine’* [with the result]. *We were very fortunate, we’ve had pretty easy lives, so we just assumed everything would be fine. Now I think back and think we were idiots.”* – Paige, increased chance, currently trying to conceive via IVF with PGT-M

*“I felt that the world was ending. All I ever wanted to be in life was to be a Mum. My husband, all he ever wants to be is a Dad, and we both grew up with wonderful families and we wanted to create our own.”* – Marnie, increased chance, planning to use PND after becoming pregnant

Given this profound impact, participants appreciated prompt access to genetic counselling to learn more about the condition and discuss their reproductive options. For many, genetic counselling appeared to be the first step towards regaining a sense of confidence in their reproductive futures. Compared to Adrian’s initial reaction to the result, his quote below demonstrates a turning point in his emotions as he started considering reproductive options with his partner.

*“…after that* [genetic counselling] *appointment was when it* [the result] *started feeling overall like a positive…it still wasn’t like ‘everything’s fantastic’*, [but] *at least we know where we’re going now.”* – Adrian, increased chance, planning to conceive via IVF with PGT-M

***Theme 2 –*** **Constructing a new reproductive path is an evolving process**

Making a reproductive decision appeared to be a key step in participants’ adaptation to their increased chance result. Several participants used the word “journey” to describe the reproductive decision-making process. The first decision participants described making was whether the condition they received an increased chance result for is one they want to avoid in their future children.

Most participants made the choice to try to avoid the condition in their children. Participants who were pregnant at result disclosure made a choice about PND quickly (quote 3), understandable given the limited time in which to do so. In comparison, those who were pre-conception generally described a more evolving decision-making process. Participants like Andrew and Joel felt that they came to a decision with their partners quickly. Others, such as partners Felicity and Felix, described how their decision was made over a series of conversations. Some also involved significant people in their lives, such as friends, family (quote 4) or religious leaders.

Most reported discussing options such as adoption or donor gametes with their genetic counsellor, however, all participants only considered IVF with PGT-M and PND. Factors involved in the decision are described in Supplementary Table 3. While IVF with PGT-M was the preferred choice for many, several participants acknowledged psychosocial, medical and practical burdens associated with IVF. Some also noted the cognitive dissonance of pursuing IVF without the prior experience of infertility. However, Felicity felt more disappointment at the thought of IVF than her partner, Felix (also interviewed):

*“…I remember feeling a bit upset that we would end up going down the IVF route before we’d even started trying* [to conceive]*.”* – Felicity, increased chance, planning to conceive via IVF with PGT-M

Two participants (Leah and Samira) became pregnant post-result and chose not to have PND. Some genes screened in Mackenzie’s Mission were associated with conditions for which early treatment can improve prognosis. Leah received an increased chance for one such condition, which she described as *“mild”* and *“manageable”*. She explained that the result therefore did not change her plans to conceive and ultimately had a less profound impact on her reproductive plans (quote 5).

At 12 months post-result, all but one participant (Tilly) who had RGCS pre-conception had made a reproductive decision. Tilly explained that she felt ambivalent toward both PND and IVF with PGT-M (quote 6). For this reason, she said: “[we’re] *not ‘not trying’ for a child, but we’re also still exploring IVF.”*

Participants who had had a pregnancy during the study period also noted that reproductive decisions are not fixed and may change for future pregnancies. For example, some participants who conceived spontaneously and had unaffected pregnancies were considering whether they are willing to attempt this option again in the future knowing that there was 25% chance that the pregnancy would be affected.

*“…it’s a tough one because obviously it was easy for us to get pregnant but then you don’t want to, again, risk that one in four chance…You are lucky this time but are you always going to be as lucky?”* – Gabby, increased chance, pregnant post-result, had PND and pregnancy was unaffected

***Theme 3 –*** **Embarking on a new reproductive path is emotionally complex**

By 12 months post-result, participants were at various stages of enacting their reproductive decisions. The following three sub-themes describe the emotional impacts of participants’ chosen reproductive pathway.

***“We’d regained our pregnancy”*** **– Experiences of PND**

Participants who had PND all described a peak in their anxiety and difficulties connecting with their pregnancy whilst waiting for the diagnostic result (quote 7). Mara used the word *“limbo”* to describe this experience. Most participants explained that their anxiety quickly resolved after learning their pregnancy was unaffected. However, some participants expressed ongoing psychological impacts after PND (quote 8). To address this, Gabby suggested: *“…more support within the pregnancy would have been good.”*

***“Half enjoying and half worrying”*** **– Experiences of pregnancy without PND**

Leah and Samira explained that they would not consider termination of pregnancy and did not have PND after becoming pregnant. Leah, who was pregnant at the time of interview, was confident in the management options should her baby have the condition. Conversely, Samira had experienced infertility and was due to begin IVF with PGT-M when she conceived spontaneously. She described the mixed emotions she experienced during pregnancy (quote 9). She also described the extremely difficult wait for the baby’s test result after birth and how relieved she was to learn her child did not have the genetic condition.

***“The uncertainty of IVF”*** **– Experiences of IVF with PGT-M**

Experiences of IVF with PGT-M varied greatly. Partners Ruby and Rory (both interviewed), and Carlos and his wife (not interviewed), conceived after their first embryo transfer. All three participants were positive about their experience with IVF and PGT-M, although Rory and Carlos noted that they had not had the embodied experience of IVF and that their role was primarily to support their partners (quote 10).

Ruby acknowledged that while she had a positive experience with IVF and PGT-M, not everyone would have the same experience. This was clear from the experiences of partners Paige and Patrick (both interviewed). Paige explained: “[I] *went into the IVF process with an expectation that it was going to work right away…”* However, the couple had experienced multiple unsuccessful embryo transfers and ovarian hyper-stimulation and at the time of interview, felt at a crossroad of how to proceed.

*“I’m looking for the quickest option* [to become pregnant]. *I’m like ‘this is all taking too long, this is really hard on my body’… the thought of going through that again* [ovarian hyper-stimulation], *I don’t want to do that.”* – Paige, increased chance, currently trying to conceive via IVF with PGT-M

*“We’ve talked about it* [stopping IVF], *but…if we did do that and got pregnant naturally, we’d still want to test for this condition and…would probably terminate the pregnancy…which would be kind of traumatic… we want to avoid that.”* - Patrick, increased chance, currently trying to conceive via IVF with PGT-M

Several participants were soon to commence their first IVF cycle. Whilst some felt excited and hopeful at the prospect, others described anticipatory anxiety and were approaching their first cycle with caution (quote 11).

***Theme 4 –*** **An increased chance result has value in the reproductive journey**

As participants made and enacted reproductive choices, the meaning ascribed to their increased chance result evolved. At 12 months post-result, many participants felt grateful for the result and did not regret the decision to have RGCS. For example, Andrew explained that it was better to find out pre-conception, rather than after the birth of a child with the condition. As such, when considering the different directions their lives may have taken had they not had RGCS, most concluded that it was better to know than not know.

*“It was 100% the right decision* [to have RGCS]*. I think it still brings a lot of trauma and upset and heartache to make* [reproductive] *decisions, but it saves a whole lot more than having a potential one in four pregnancy* [with the condition]*.”* – Tilly, increased chance, currently deciding on a reproductive option

Nevertheless, the emotional complexities of receiving an increased chance result appear to be long-lasting. Two participants, who were pregnant at result disclosure and had unaffected pregnancies, have chosen not to have more children. Although they had reached the end of their reproductive journey, both described ongoing emotional impacts of their result. Vera, for example, explained how she and her husband feared an unexpected pregnancy (quote 12) as they do not want to experience the stress of PND and possible termination of pregnancy a second time.

Additionally, participants who were trying to conceive, or were planning a future pregnancy, described the result remaining at the forefront of their minds. Further, the steps involved in IVF have meant some participants are considering a future pregnancy sooner than they may have otherwise (quote 13). As Samira said: “*It’s hard…I don’t think I could not think about it…*”

## Discussion

This study describes experiences of RGCS within the first 12 months post-result. Strengths of our study included the large sample size and the longitudinal, mixed-methods design, which enabled us to comprehensively examine psychosocial and reproductive impacts. For most people, decision regret was minimal and reproductive confidence was high, although those with an increased chance result had elevated levels of anxiety compared to baseline. Interviews provided rich explanations for these survey results by highlighting the emotional complexities associated with receiving an increased chance result. The evidence generated in this study can be used to inform how best to deliver RGCS in a way that promotes positive outcomes.

Most people who pursue RGCS will receive a low chance result [1]. Our findings emphasise that this experience has minimal negative psychosocial implications and provides reproductive confidence within the first 12 months post-result. Levels of anxiety were in line with population norms [29] and remained relatively stable from enrolment to 12 months post-result. Similar results have been reported up to six months post-result [25, 30]. The large sample size of this study, along with previously reported smaller studies, indicate that RGCS can be delivered at a population-level without causing harm to most who take part [25, 30].

Thus, whilst the act of being offered RGCS does not appear to adversely impact anxiety, receipt of an increased chance result is psychosocially impactful. Several qualitative studies have described emotional responses such as shock and distress in the days and weeks following result disclosure [8–11]. By 12 months post-result, we observed levels of anxiety at or almost at clinically meaningful levels for participants with new and known increased chance results. Similar levels of anxiety were observed in Mackenzie’s Mission at three months post-result [1]. Our results therefore show that the emotional impact of an increased chance result can persist. This contrasts with research in the context of predictive genetic testing (e.g., for Huntington disease), in which any increased anxiety typically resolves within the first year post-result [31].

The Reproductive Story can provide a conceptual basis to interpreting these findings. A ‘reproductive story’ refers to a narrative a person creates about what it will be like to be a parent [32]. The development of the reproductive story begins in childhood and is shaped by factors such as life experience, the family system, and social and cultural norms [32]. These factors influence expectations around a person’s future reproductive journey [32]. When these expectations are not met, the reproductive story becomes threatened [33]. The term ‘reproductive trauma’ refers to any reproductive event that negatively impacts the reproductive story, such as infertility and perinatal loss [32]. Evidence of reproductive trauma has also been observed amongst reciprocal translocation carriers and their partners [34].

Our findings show that an increased chance result disrupts the reproductive story and can be a form of reproductive trauma [32]. Participants in several RGCS studies, including ours, have described grieving the loss of the expected reproductive path after receipt of an increased chance result [35]. In our study, grief was most apparent for participants using IVF in the absence of known fertility problems. Given the lengthy PGT-M process, participants also described needing to adjust their expectations around when they would conceive. Grieving lost time and the spontaneity of conception has been reported in similar studies, although the magnitude and duration of grief responses post-RGCS have yet to be explored [18, 35].

An important aspect of the Reproductive Story is that a person’s story can be re-written after a reproductive trauma [32]. Our study demonstrates how people begin to revise their reproductive story in the first 12 months post-result by making reproductive decisions. Our findings regarding the relative advantages and disadvantages of IVF with PGT-M or PND reflect what is already known about the complexities of reproductive decision-making [13]. No participants interviewed had considered the use of donor gametes and further research could explore whether attitudes toward this reproductive option change during the reproductive journey. It is also important to note, however, that in Mackenzie’s Mission, couples were offered one funded cycle of IVF with PGT-M. In Australia, and most parts of the world, IVF and PGT-M incur significant expenses which may prevent people from accessing the reproductive option that they most prefer and/or is most value-consistent [14]. Incorporating funding for IVF and PGT-M into RGCS programs would increase equity of access and may also reduce some of the psychosocial impacts associated reproductive decision-making [14].

Our findings also demonstrate that the process of revising the reproductive story is not always straightforward. Whilst 41% of survey respondents with a new increased chance result reported high reproductive confidence, interview findings indicate that this confidence may fluctuate. As people embark on a new reproductive path, additional reproductive traumas may be experienced [32]. This was most markedly observed in the experiences of Paige and Patrick, who described increasing distress with each unsuccessful round of IVF, with the psychosocial impact in part due to their lack of experience with infertility and therefore higher expectations of pregnancy. Further, most of our survey and interview participants had not completed their families by 12 months post-result, meaning their reproductive stories were still evolving. This highlights the importance of RGCS research and programs continuing to capture reproductive outcomes of people with an increased chance result, to understand if and how results are useful for reproductive planning [36]. Several participants were also undecided about their future reproductive pathway and were actively engaging, or anticipating re-engaging, with the decision-making process. This may explain why elevated levels of anxiety have persisted beyond three months post-result [1], rather than reduced.

Although levels of anxiety were elevated, survey respondents who received an increased chance result reported minimal regret about the decision to pursue RGCS. Interviewees similarly described empowerment arising from the reproductive autonomy afforded by their result. Participants in prior studies also generally agreed that the benefits of knowing about an increased chance result outweighed the potential psychological burdens [8, 10, 18]. These findings suggest that, with time, RGCS results add considerable value to people’s reproductive stories.

### Implications for practice

Another important consideration in the ongoing implementation of RGCS is how best to support people who receive an increased chance result throughout their reproductive journey. Whilst elevated anxiety is somewhat expected and suggests participants had understood the implications of the result, our findings do highlight a need for long-term support. The value of genetic counselling is well established [15, 18, 37] and practice resources also emphasise the importance of post-result counselling being available [3]. Participants in our study described moments in the first 12 months post-result in which re-engagement with a genetic counsellor may be most beneficial (e.g., deciding whether to continue with IVF), but longer-term research is needed to comprehensively map when, and what, support needs arise over time. Building this evidence-base will inform how to deliver an effective and feasible model of post-result support.

### Limitations

Our sample contained an over-representation of people with a tertiary education. Response bias also means that not all experiences were captured. For example, no participant with an increased chance who had a pregnancy affected by the genetic condition responded to the interview invitation. As such, we cannot describe the impact that continuing the pregnancy or having a pregnancy termination has on the reproductive story. Based on prior research however, it is likely that these experiences have significant, and distinctive, psychosocial impacts [9] that warrant examination.

## Conclusion

Our study described psychosocial and reproductive impacts of RGCS within the first 12 months post-result within a large research cohort. Most participants had minimal decision regret and high reproductive confidence. However, receiving an increased chance result disrupts people’s reproductive story and as such, anxiety was elevated. By 12 months post-result, participants were at various stages of revising their reproductive story and described finding value in the information from RGCS, given the reproductive empowerment it provided. Using the concept of the Reproductive Story provided a unique lens through which to examine how people navigate increased chance results over time, and further research is now planned to understand how reproductive stories evolve longer-term.

## Supplementary information

Supplementary Table 1

Supplementary Table 2

Supplmentary Table 3

## Data Availability

De-identified data that support the findings of this study are available from the corresponding author upon request.

## References

[1] Kirk E, Delatycki MB, Archibald AD, Tutty E, Caruana J, Halliday JL, et al. Nationwide, couple-based genetic carrier screening. N Engl J Med. 2024;391:1877–89. 39565987 10.1056/NEJMoa2314768

[2] The Royal Australian College of General Practitioners. Genomics in general practice. East Melbourne, Victoria: The Royal Australian College of General Practitioners; 2018. https://www.racgp.org.au/getattachment/63568f23-e288-4a0e-a23a-39fbd046cc21/Genomics-in-general-practice.aspx.

[3] Gregg AR, Aarabi M, Klugman S, Leach NT, Bashford MT, Goldwaser T, et al. Screening for autosomal recessive and X-linked conditions during pregnancy and preconception: a practice resource of the American College of Medical Genetics and Genomics (ACMG). Genet Med. 2021;23:1793–806. 34285390 10.1038/s41436-021-01203-z PMC8488021

[4] The Royal Australian and New Zealand College of Obstetricians and Gynaecologists. C-Obs 63 Genetic Carrier Screening Clinical Guidance Statement. 2019. https://www.rcpa.edu.au/getattachment/768dc44f-618c-4934-89da-6b22c446c240/Guidelines-for-reproductive-geneticcarrier-screen.aspx.

[5] The Royal College of Pathologists of Australasia, Human Genetics Society of Australasia. Guidelines for reproductive genetic carrier screening for cystic fibrosis, fragile X syndrome and spinal muscular atrophy. 2024. 10.1038/ejhg.2015.271.

[6] Henneman L, Borry P, Chokoshvili D, Cornel MC, van El CG, Forzano F, et al. Responsible implementation of expanded carrier screening. Eur J Hum Genet. 2016;24:e1–e12. 10.1038/ejhg.2015.271 PMC4867464 26980105

[7] Van Steijvoort E, Cassou M, De Schutter C, Dimitriadou E, Peeters H, Peeraer K, et al. Exploring attitudes and experiences with reproductive genetic carrier screening among couples seeking medically assisted reproduction: a longitudinal survey study. J Assist Reprod Genet. 2024:41:451–64. 10.1007/s10815-023-03010-8 PMC10894802 38175314

[8] Tardif J, Pratte A, Laberge A-M. Experience of carrier couples identified through a population-based carrier screening pilot program for four founder autosomal recessive diseases in Saguenay-Lac-Saint-Jean. Prenat Diagn. 2018;38:67–74. 28419508 10.1002/pd.5055

[9] Ioannou L, Delatycki MB, Massie J, Hodgson J, Lewis S. Suddenly having two positive people who are carriers is a whole new thing” - experiences of couples both identified as carriers of cystic fibrosis through a population-based carrier screening program in Australia. J Genet Couns. 2015;24:987–1000. 25925605 10.1007/s10897-015-9833-9

[10] Beard CA, Amor DJ, Di Pietro L, Archibald AD. “I’m Healthy, It’s Not Going To Be Me”: exploring experiences of carriers identified through a population reproductive genetic carrier screening panel in Australia. Am J Med Genet. 2016;170:2052–9. 27150953 10.1002/ajmg.a.37697

[11] Mathijssen IB, Holtkamp KCA, Ottenheim CPE, van Eeten-Nijman JMC, Lakeman P, Meijers-Heijboer H, et al. Preconception carrier screening for multiple disorders: evaluation of a screening offer in a Dutch founder population. Eur J Hum Genet. 2018;26:166–75. 29321671 10.1038/s41431-017-0056-4 PMC5838981

[12] Cannon J, Van Steijvoort E, Borry P, Chokoshvili D. How does carrier status for recessive disorders influence reproductive decisions? A systematic review of the literature. Expert Rev Mol Diagn. 2019;19:1117–29. 31709839 10.1080/14737159.2020.1690456

[13] Severijns Y, de Die-Smulders CEM, Gültzow T, de Vries H, van Osch LADM. Hereditary diseases and child wish: exploring motives, considerations, and the (joint) decision-making process of genetically at-risk couples. J Community Genet. 2021;12:325–35. 33611773 10.1007/s12687-021-00510-x PMC8241960

[14] Delatycki MB, Alkuraya F, Archibald A, Castellani C, Cornel M, Grody WW, et al. International perspectives on the implementation of reproductive carrier screening. Prenat Diagn. 2020;40:301–10. 31774570 10.1002/pd.5611

[15] Archibald AD, Smith MJ, Burgess T, Scarff KL, Elliott J, Hunt CE, et al. Reproductive genetic carrier screening for cystic fibrosis, fragile X syndrome, and spinal muscular atrophy in Australia: outcomes of 12,000 tests. Genet Med. 2018;20:513–23. 29261177 10.1038/gim.2017.134

[16] Johansen Taber KA, Beauchamp KA, Lazarin GA, Muzzey D, Arjunan A, Goldberg JD. Clinical utility of expanded carrier screening: results-guided actionability and outcomes. Obstet Gynecol Surv. 2019;74:582–4. 10.1038/s41436-018-0321-0 PMC6752268 30310157

[17] Ghiossi CE, Goldberg JD, Haque IS, Lazarin GA, Wong KK. Clinical utility of expanded carrier screening: reproductive behaviors of at-risk couples. J Genet Couns. 2018;27:616–25. 28956228 10.1007/s10897-017-0160-1 PMC5943379

[18] Richardson E, McEwen A, Newton-John T, Crook A, Jacobs C. Outcomes of importance to patients in reproductive genetic carrier screening: a qualitative study to inform a core outcome set. J Pers Med. 2022;12:1310. 10.3390/jpm12081310 PMC9409855 36013258

[19] Richardson E, McEwen A, Newton-John T, Jacobs C. Defining core outcomes of reproductive genetic carrier screening: a Delphi survey of Australian and New Zealand stakeholders. Prenat Diagn. 2023;43:1150–65. 37526246 10.1002/pd.6410

[20] Richardson E, McEwen A, Newton-John T, Crook A, Jacobs C. Systematic review of outcomes in studies of reproductive genetic carrier screening: towards development of a core outcome set. Genet Med. 2022;24:1–14. 34906455 10.1016/j.gim.2021.08.005

[21] Archibald AD, McClaren BJ, Caruana J, Tutty E, King EA, Halliday JL, et al. The Australian reproductive genetic carrier screening project (Mackenzie’s Mission): design and implementation. J Pers Med. 2022;12:1781. 36579509 10.3390/jpm12111781 PMC9698511

[22] Creswell JW, Plano Clark VL. Choosing a mixed methods design. Designing Conducting Mixed Methods Res. 2011;2:53–106.

[23] Thorne S. Interpretive description: qualitative research for applied practice. New York: Routledge; 2016.

[24] Marteau TM, Bekker H. The development of a six-item short-form of the state scale of the Spielberger State—Trait Anxiety Inventory (STAI). Br J Clin Psychol. 1992;31:301–6. 1393159 10.1111/j.2044-8260.1992.tb00997.x

[25] Birnie E, Schuurmans J, Plantinga M, Abbott KM, Fenwick A, Lucassen A, et al. Couple-based expanded carrier screening provided by general practitioners to couples in the Dutch general population: psychological outcomes and reproductive intentions. Genet Med. 2021;23:1761–8. 34112999 10.1038/s41436-021-01199-6 PMC8460434

[26] Brehaut JC, O’Connor AM, Wood TJ, Hack TF, Siminoff L, Gordon E, et al. Validation of a decision regret scale. Medical Decis Mak. 2003;23:281–92. 10.1177/0272989X03256005 12926578

[27] Palinkas LA, Horwitz SM, Green CA, Wisdom JP, Duan N, Hoagwood K. Purposeful sampling for qualitative data collection and analysis in mixed method implementation research. Adm Policy Ment Health. 2015;42:533–44. 24193818 10.1007/s10488-013-0528-y PMC4012002

[28] Braun V, Clarke V, Hayfield N, Davey L, Jenkinson E. Doing reflexive thematic analysis. In: Bager-Charleson S, McBeath A, editors. Supporting research in counselling and psychotherapy: qualitative, quantitative, and mixed methods research. Cham: Springer International Publishing; 2022.

[29] Crawford J, Cayley C, Lovibond PF, Wilson PH, Hartley C. Percentile norms and accompanying interval estimates from an Australian general adult population sample for self-report mood scales (BAI, BDI, CRSD, CES-D, DASS, DASS-21, STAI-X, STAI-Y, SRDS, and SRAS). Aust Psychologist. 2011;46:3–14.

[30] van Dijke I, Lakeman P, Sabiri N, Rusticus H, Ottenheim CPE, Mathijssen IB, et al. Couples’ experiences with expanded carrier screening: evaluation of a university hospital screening offer. Eur J Hum Genet. 2021;29:1252–8. 34155360 10.1038/s41431-021-00923-9 PMC8384865

[31] Broadstock M, Michie S, Marteau T. Psychological consequences of predictive genetic testing: a systematic review. Eur J Hum Genet. 2000;8:731–8. 11039571 10.1038/sj.ejhg.5200532

[32] Jaffe J. Reproductive trauma: psychotherapy with clients experiencing infertility and pregnancy loss. Washington, DC, US: American Psychological Association; 2024.

[33] Jaffe J. The reproductive story: Parents’ possible selves and how things should have been. Reproductive trauma: psychotherapy with clients experiencing infertility and pregnancy loss, 2nd ed. Washington, DC, US: American Psychological Association; 2024.

[34] Cifuentes Ochoa M, Flowers NJ, Pertile MD, Archibald AD. “It becomes your whole life”—exploring experiences of reciprocal translocation carriers and their partners. J Genet Couns. 2023;32:1057–68. 37186486 10.1002/jgc4.1716

[35] Richardson E, McEwen A, Newton-John T, Crook A, Jacobs C. Incorporating patient perspectives in the development of a core outcome set for reproductive genetic carrier screening: a sequential systematic review. Eur J Hum Genet. 2022;30:756–65. 35347269 10.1038/s41431-022-01090-1 PMC9259674

[36] Henneman L. Genetic carrier screening — call for a global mission. New Engl J Med. 2024;391:1947–8. 39565994 10.1056/NEJMe2410086

[37] Edwards S, Laing N. Genetic counselling needs for reproductive genetic carrier screening: a scoping review. J Pers Med. 2022;12:1699. 36294838 10.3390/jpm12101699 PMC9605645

