# Supplementary material for: Revising the reproductive story: psychosocial and reproductive impacts 12 months after reproductive genetic carrier screening
Source: Eur J Hum Genet. 2025 Jul 9;33(8):1035–43. doi: 10.1038/s41431-025-01903-z (PMC12322158; doi:10.1038/s41431-025-01903-z)
Supplement: Supplementary file 1 — Supplementary Table 1 [file 41431_2025_1903_MOESM1_ESM.docx]

**Supplementary Table 1. Characteristics of responders compared with non-responders to the 12 month post-result survey using ꓫ^2^ tests**

|  | **Responders**  **(N=4164)** | **Non-responders**  **(N=4889)** | ***p* value** |
| --- | --- | --- | --- |
| **Couple data^a^ (N, %)** |  |  |  |
| *Recruitment source* |  |  | p<0.001 |
| General practice | 1938 (46.5) | 2129 (43.5) |  |
| Fertility clinic | 485 (11.6) | 733 (14.9) |  |
| Genetics clinic | 642 (15.4) | 679 (13.8) |  |
| Private obstetrics | 678 (16.2) | 912 (18.6) |  |
| Public obstetrics | 421 (10.1) | 436 (8.9) |  |
| *Geographic location* |  |  | p=0.002 |
| Major city | 3404 (81.7) | 3858 (78.9) |  |
| Inner regional | 502 (12) | 653 (13.3) |  |
| Outer regional | 218 (5.2) | 333 (6.8) |  |
| Remote/Very remote | 40 (0.9) | 45 (0.9) |  |
| *IRSAD quintile by SA2* |  |  | p<0.001 |
| Quintile 1 | 251 (6) | 506 (10.3) |  |
| Quintile 2 | 552 (13.2) | 711 (14.5) |  |
| Quintile 3 | 779 (18.7) | 982 (20) |  |
| Quintile 4 | 991 (23.7) | 1185 (24.2) |  |
| Quintile 5 | 1591 (38.2) | 1505 (30.7) |  |
| *Relationship status* |  |  | p<0.001 |
| Married | 2525 (60.6) | 2883 (58.9) |  |
| Living with partner | 1567 (37.6) | 1854 (37.9) |  |
| Not living with partner | 66 (1.5) | 134 (2.7) |  |
| Other | 6 (0.1) | 18 (0.3) |  |
| *Language spoken at home* |  |  | p<0.001 |
| English only | 3696 (88.7) | 3990 (81.6) |  |
| English and other language(s) | 349 (8.3) | 554 (11.3) |  |
| Language(s) other than English | 119 (2.8) | 345 (7) |  |
| *Annual household income* |  |  | p<0.001 |
| Up to $50,000 | 60 (1.4) | 189 (3.8) |  |
| $50,001-$100,000 | 426 (10.2) | 820 (16.7) |  |
| $100,001-$150,000 | 958 (23) | 1146 (23.4) |  |
| $150,001-$200,000 | 1098 (26.3) | 1111 (22.7) |  |
| $200,001+ | 1328 (31.8) | 1020 (20.8) |  |
| Prefer not to say | 294 (7) | 596 (12.2) |  |
| *Relevant family history of a genetic condition^c^* |  |  | p=0.495 |
| No | 3765 (90.4) | 4441 (90.8) |  |
| Yes | 399 (9.5) | 448 (9.1) |  |
| *Consanguineous couple* |  |  | p<0.001 |
| No | 4149 (99.6) | 4822 (98.6) |  |
| Yes | 15 (0.3) | 67 (1.3) |  |
| *Pregnant at enrolment* |  |  | p<0.001 |
| No | 3473 (83.4) | 3864 (79) |  |
| Yes | 691 (16.5) | 1025 (20.9) |  |
| *Number of children* |  |  | p<0.001 |
| Zero | 3122 (74.9) | 3447 (70.5) |  |
| One | 783 (18.8) | 1054 (21.5) |  |
| Two or greater | 259 (6.2) | 388 (7.9) |  |
| *Has a child a with medical condition/disability* |  |  | p=0.001 |
| No | 3930 (94.3) | 4531 (92.6) |  |
| Yes | 234 (5.6) | 358 (7.3) |  |
| *Experienced a miscarriage* |  |  | p<0.001 |
| No | 3332 (80) | 3754 (76.7) |  |
| Yes | 832 (19.9) | 1135 (23.2) |  |
| *Experienced a stillbirth* |  |  | p=0.065 |
| No | 4041 (97) | 4775 (97.6) |  |
| Yes | 123 (2.9) | 114 (2.3) |  |
| *Had a termination of pregnancy* |  |  | p=0.112 |
| No | 3652 (87.7) | 4233 (86.5) |  |
| Yes | 512 (12.2) | 656 (13.4) |  |
| *Has had difficulties conceiving* |  |  | p<0.001 |
| No | 1315 (31.5) | 1745 (35.6) |  |
| Yes - Haven't used fertility treatment | 342 (8.2) | 528 (10.7) |  |
| Yes - Have used fertility treatment | 813 (19.5) | 1034 (21.1) |  |
| Haven't tried to conceive | 1694 (40.6) | 1582 (32.3) |  |
| *Intention to conceive in the future* |  |  | p<0.001 |
| In the next year | 2515 (60.3) | 2746 (56.1) |  |
| In more than one year | 1174 (28.1) | 1449 (29.6) |  |
| Unsure | 367 (8.8) | 517 (10.5) |  |
| No plans | 108 (2.5) | 177 (3.6) |  |
| **Individual data^c^ (N, %)** | **Responders**  **(N=4984)** | **Non-responders** | ***p* value** |
|  |  | **(N=13,120)** |  |
| *Gender* |  |  | p<0.001 |
| Female | 3562 (71.4) | 5517 (42) |  |
| Male | 1419 (28.4) | 7593 (57.8) |  |
| Gender diverse | 0 (0) | 4 (0) |  |
| Prefer not to say | 3 (0) | 6 (0) |  |
| *Age category (years)* |  |  | p<0.001 |
| Under 25 | 52 (1) | 277 (2.1) |  |
| 25-29 | 741 (14.8) | 2311 (17.6) |  |
| 30-34 | 2246 (45) | 5183 (39.5) |  |
| 35-39 | 1508 (30.2) | 3773 (28.7) |  |
| 40 or over | 437 (8.7) | 1577 (12) |  |
| *Born in Australia* |  |  | p<0.001 |
| No | 1145 (22.9) | 3511 (26.7) |  |
| Yes | 3839 (77) | 9610 (73.2) |  |
| *Aboriginal or Torres Strait Islander descent* |  |  | p=0.007 |
| No | 4946 (99.2) | 12959 (0) |  |
| Yes | 38 (0.7) | 162 (0) |  |
| *Ancestry^d^* |  |  |  |
| Northern or Western Europe | 3547 (71.1) | 7506 (56.8) | p<0.001 |
| Southern Europe | 715 (14.3) | 1840 (13.9) | p=0.353 |
| Eastern Europe | 385 (7.7) | 828 (6.2) | p=0.017 |
| Oceania | 346 (6.9) | 1083 (8.1) | p<0.001 |
| Asia | 636 (12.7) | 2062 (15.6) | p<0.001 |
| Middle East | 120 (2.4) | 461 (3.4) | p<0.001 |
| Africa | 90 (1.8) | 276 (2) | p=0.076 |
| North America | 26 (0.5) | 64 (0.4) | p=0.976 |
| Central or South America | 77 (1.5) | 208 (1.5) | p=0.515 |
| Unknown | 310 (6.2) | 1540 (11.6) | p<0.001 |
| *Religious affinity* |  |  | p<0.001 |
| No religion | 2970 (59.5) | 7089 (54) |  |
| Buddhism | 82 (1.6) | 249 (1.8) |  |
| Christianity | 1652 (33.1) | 4551 (34.6) |  |
| Hinduism | 70 (1.4) | 360 (2.7) |  |
| Islam | 48 (0.9) | 351 (2.6) |  |
| Judaism | 89 (1.7) | 180 (1.3) |  |
| Other religion | 23 (0.4) | 122 (0.9) |  |
| Prefer not to say | 50 (1) | 218 (1.6) |  |
| *Influence of religion on life decisions* |  |  | p<0.001 |
| Not at all | 3498 (70.1) | 8704 (66.3) |  |
| Very little | 762 (15.2) | 2087 (15.9) |  |
| Moderately | 500 (10) | 1461 (11.1) |  |
| Very much | 158 (3.1) | 572 (4.3) |  |
| Completely | 66 (1.3) | 297 (2.2) |  |
| *Perceived financial situation* |  |  | p<0.001 |
| Very difficult | 5 (0.1) | 18 (0.1) |  |
| Quite difficult | 16 (0.3) | 74 (0.5) |  |
| Getting by | 186 (3.7) | 795 (6) |  |
| Comfortable | 2554 (51.2) | 5681 (43.3) |  |
| Doing all right | 2111 (42.3) | 5945 (45.3) |  |
| Prefer not to say | 112 (2.2) | 589 (4.4) |  |
| *Highest level of education attained* |  |  | p<0.001 |
| Bachelor degree or above | 3958 (79.4) | 7616 (58) |  |
| Advanced diploma or diploma | 123 (2.4) | 377 (2.8) |  |
| Certificate | 597 (11.9) | 3233 (24.6) |  |
| High school or below | 306 (6.1) | 1892 (14.4) |  |
| *(missing)* |  | 3 (0) |  |
| *Employment status* |  |  | p<0.001 |
| Full time | 3820 (76.6) | 10637 (81) |  |
| Part time, casual or contract work | 890 (17.8) | 1708 (13) |  |
| Home duties | 132 (2.6) | 348 (2.6) |  |
| Student | 72 (1.4) | 139 (1) |  |
| Unemployed/unable to work | 70 (1.4) | 268 (2) |  |
| *Perceived family history of a condition that is, or might be, genetic* |  |  | p<0.001 |
| No | 2485 (49.8) | 6497 (49.5) |  |
| Yes | 1306 (26.2) | 2852 (21.7) |  |
| Unsure | 1193 (23.9) | 3772 (28.7) |  |

^a^ The number of responding and non-responding couples does not match the total number of reproductive couples in Mackenzie’s Mission (N=9107) for two reasons. First, n=49 responders were excluded because they reported in the 12 month post-result survey that they had separated or were now using a gamete donor to conceive after receiving a low chance result with their partner. Second, when analysing couple-specific variables, data pertaining to the individuals/pairs who would be raising the child was used (N=9102)^1^ meaning five non-responder reproductive couples were excluded (four sex couples where both partners had reproductive genetic carrier screening with the same gamete donor and one couple where one participant received a low chance with their partner and subsequently a gamete donor).

^b^ The couple had a family history of a genetic condition screened in Mackenzie’s Mission.

^c^ The number of responding and non-responding participants does not match the total number of participants in Mackenzie’s Mission (N=18,219); n=53 responders were excluded because they had separated or were now using a gamete donor to conceive after receiving a low chance result with their partner, n=19 were excluded because they were a gamete donor and were not required to complete additional research activities^1,20^ and n=43 participants were excluded because they opted-out additional research activities after enrollment and were thus not invited to complete the survey.

^d^ Participants could select ‘Yes’ to more than one ancestry variable. Percentages and *p* values are reported per variable.
