# Supplementary material for: Revising the reproductive story: psychosocial and reproductive impacts 12 months after reproductive genetic carrier screening
Source: Eur J Hum Genet. 2025 Jul 9;33(8):1035–43. doi: 10.1038/s41431-025-01903-z (PMC12322158; doi:10.1038/s41431-025-01903-z)
Supplement: Supplementary file 2 — Supplementary Table 2 [file 41431_2025_1903_MOESM2_ESM.docx]

**Supplementary Table 2. Psychosocial and reproductive outcomes 12 months after receiving an increased chance result**

|  | **A**  **Planning first pregnancy post- result** | **B**  **Currently pregnant** | **C**  **Baby born & family complete** | **D**  **Baby born & planning a pregnancy** | **E**  **Pregnancy loss & planning a pregnancy** | **E**  **Unsure about planning a pregnancy** |
| --- | --- | --- | --- | --- | --- | --- |
| **Couples (N)^a^** | 31 | 23 | 12 | 16 | 6 | 4 |
| **Timing of pregnancy** |  |  |  |  |  |  |
| Before results |  |  | 9 | 16 | 1 | 1 |
| After results |  | 23 | 3 |  | 5 | 2 |
| No pregnancy | 31 |  |  |  |  | 1 |
| **Reproductive choice for first pregnancy** |  |  |  |  |  |  |
| IVF with PGT-M |  | 14 | 1 |  | 3^h^ |  |
| PND, TOP if affected |  | 3^c^ | 7^e^ | 10 | 3^i^ | 2^k^ |
| Plan and prepare |  | 6 | 4^f^ | 5^g^ |  |  |
| Not stated |  |  |  | 1 |  | 1 |
| **Current or future reproductive plans** |  |  |  |  |  |  |
| IVF with PGT-M | 20 | 10 |  | 2 | 2 |  |
| PND | 2 | 2 |  | 4 | 3 |  |
| Plan and prepare |  | 4 |  | 2 |  |  |
| Undecided | 9^b^ | 5^d^ |  | 8^d^ | 1^j^ | 4^l^ |
| Not planning a future pregnancy |  | 2 | 12 |  |  |  |
| **Individuals (N)** | 35 | 32 | 14 | 17 | 8 | 6 |
| Anxiety at enrolment | 30 (23.3-40) | 26.7 (20-36.7) | 36.7 (30.0-43.3) | 33.3 (26.7-36.7) | 41.7 (28.3-50) | 26.7 (26.7-26.7) |
| Anxiety at 12 months post-result | 40 (30-53.3) | 35 (23.3-41.7) | 35 (26.7-53.3) | 40 (26.7-56.7) | 51.7 (43.3-61.7) | 50 (33.3-53.3) |
| Anxiety at 12 months post-result ≥40 | 19 (54.3%) | 12 (37.5%) | 6 (42.9%) | 9 (52.9%) | 8 (100%) | 3 (60.0%) |
| Decision regret | 5 (0-15) | 0 (0-7.5) | 12.5 (0-20) | 0 (0-10) | 10 (0-12.5) | 15 (15-30) |
| **Reproductive confidence** |  |  |  |  |  |  |
| Not much confidence | 3 (8.6%) | 2 (6.3%) | 1 (7.1%) | 1 (5.9%) | 1 (12.5%) | 1 (20.0%) |
| Some confidence | 21 (60.0%) | 13 (40.6%) | 5 (35.7%) | 8 (47.1%) | 6 (75.0%) | 3 (60.0%) |
| A lot of confidence | 11 (31.4%) | 17 (53.1%) | 8 (57.1%) | 8 (47.1%) | 1 (12.5%) | 1 (20.0%) |

IVF with PGT-M = in vitro fertilisation with pre-implantation genetic testing for the monogenic condition | PND= prenatal diagnosis with the view to end an affected pregnancy |Median (interquartile range) anxiety as measured by the 6-item State Trait Anxiety Inventory (score range 20-80, higher scores indicate higher anxiety)^29^ with scores ≥40 indicate clinically meaningful anxiety.^30^ | Median (interquartile range) decision regret as measured by the Decision Regret Scale (score range 0-100, 0=no regret, 100=high regret).^31^

^a^ N=92 couples. Six couples did not provide sufficient reproductive outcomes data to classify them into groups A – E. ^b^ Includes four couples trying spontaneous conception and IVF with PGT-M, two couples who have tried IVF with PGT-M and are now re-considering their plans and two couples who are considering IVF with PGT-M but have not decided. ^c^ Two pregnancies were unaffected, and the PND result was pending at the time of survey completion for one couple. ^d^ Undecided between IVF with PGT-M and PND. ^e^ Includes six couples were pregnant at result disclosure. All seven pregnancies were unaffected. ^f^ None of the babies are known to have the genetic condition. ^g^ Two babies were diagnosed with the genetic condition. ^h^ One couple had a termination for medical reasons unrelated to the genetic condition they had an increased chance for, two couples had a miscarriage. ^I^ One couple had an unaffected pregnancy but the reason for the pregnancy loss was not stated. Two couples had a miscarriage before PND could be performed. ^j^ Deciding between IVF with PGT-M and using donor gametes. ^j^ One pregnancy was unaffected and carried to term. One couple had an affected pregnancy and had a termination. ^k^ Three couples would consider IVF with PGT-M if they were to try to conceive in the future.
