# Supplementary material for: Revising the reproductive story: psychosocial and reproductive impacts 12 months after reproductive genetic carrier screening
Source: Eur J Hum Genet. 2025 Jul 9;33(8):1035–43. doi: 10.1038/s41431-025-01903-z (PMC12322158; doi:10.1038/s41431-025-01903-z)
Supplement: Supplementary file 3 — Supplmentary Table 3 [file 41431_2025_1903_MOESM3_ESM.docx]

**Supplementary Table 3. Factors influencing reproductive decision-making between IVF with PGT-M and prenatal diagnosis**

|  | **IVF with PGT-M** | | **Prenatal diagnosis with the view to end an affected pregnancy** | |
| --- | --- | --- | --- | --- |
| **The role of risk perception** | - A perception that a 25% chance of an affected pregnancy is *"too risky"* | | - A perception that a 75% chance of an unaffected pregnancy is greater odds than the chance of a successful pregnancy using IVF | |
|  | Perceived advantages | Perceived disadvantages | Perceived advantages | Perceived disadvantages |
| **Psychosocial factors** | - The *"safe"* choice to protect against the emotional toll of potential termination of pregnancy - Provides greater certainty in early pregnancy | - Uncertainty of if, and when, a cycle of IVF will be successful - Conception is *“not going to be spontaneous”* | - Experiencing spontaneous conception without fertility intervention (if not experiencing fertility problems) | - *“Anxiety*” during early pregnancy and while waiting for prenatal diagnosis results - The *“grief of going through termination”* of pregnancy |
| **Medical factors** |  | - The physical burdens on the partner carrying the pregnancy - Potential adverse reaction(s) to IVF medications/procedures |  | - Discomfort of an invasive diagnostic test procedure - Small chance of procedure-related miscarriage |
| **Practical factors** |  | - A time-consuming process - A *“financial investment”* - Coordinating medical appointments around work and life commitments | - May become pregnant quicker (if not experiencing fertility problems) |  |

Text in quotation marks and italics are participant quotes | IVF with PGT-M = in vitro fertilisation with pre-implantation genetic testing for the monogenic condition | Prenatal diagnosis is used to refer to the choice to become pregnant spontaneously or with IVF without the use of PGT-M and have diagnostic testing with the view to end an affected pregnancy.
